# NGF Signaling Exacerbates KOA Peripheral Hyperalgesia via the Increased TRPV1-Labeled Synovial Sensory Innervation in KOA Rats

**DOI:** 10.1155/2024/1552594

**Published:** 2024-02-19

**Authors:** Zixiu Liu, Mingchao Li, Li Zhang, Xiaoqing Shi, Taiyang Liao, Lishi Jie, Likai Yu, Peimin Wang

**Affiliations:** ^1^Jiangnan University, Wuxi 214000, China; ^2^Yunnan Baiyao Group Wuxi Pharmaceutical Co., Ltd., Wuxi 214000, China; ^3^Key Laboratory for Metabolic Diseases in Chinese Medicine, Nanjing University of Chinese Medicine, Nanjing 210023, China; ^4^Department of Orthopedics Surgery, The Third People's Hospital of Kunshan, Suzhou 215300, China; ^5^Department of Orthopedics, The Affiliated Hospital of Nanjing University of Chinese Medicine, Nanjing 210029, China

## Abstract

**Objectives:**

Knee osteoarthritis (KOA) pain is caused by nociceptors, which are actually sensory nerve fiber endings that can detect stimuli to produce and transmit pain signals, and high levels of NGF in synovial tissue led to peripheral hyperalgesia in KOA. The purpose of this study is to investigate how sensory nerve fibers respond to the NGF/TrKA signal pathway and mediate the peripheral hyperalgesia in KOA rats.

**Methods:**

Forty SD male rats were randomly divided into 4 groups: normal, KOA, KOA + NGF, and KOA + siRNA TrKA. KOA model rats were induced by anterior cruciate ligament transection (ACLT). Mechanical and cold withdrawal thresholds (MWT and CWT) were measured 4 times in each group. The synovial tissues were harvested on day 28, and the expressions of NGF, TrKA, TRPV1, IL-1*β*, and PGP9.5 were determined using western blot, qPCR, and immunofluorescence staining. The primary rat fibroblast-like synoviocytes (FLSs) and DRG cells were divided into 4 groups as in vivo. The expressions of NGF, TrKA, TRPV1, and CGRP in vitro were determined using western blot and qPCR.

**Results:**

KOA and intra-articular injection with NGF protein increased both mRNA and protein levels, not only TRPV1, PGP 9.5, and IL-1*β* in the synovial tissue, but also TRPV1, PGP 9.5, and S100 in the DRG tissue, while above changes were partly reversed after siRNA TrKA intervention. Besides, siRNA TrKA could improve peripheral hyperalgesia and decreased the TRPV1 positive nerve fiber innervation in synovial tissue. The results in vitro were consistent with those in vivo.

**Conclusion:**

This study showed the activation of the NGF/TrKA signaling pathway in KOA promoted the release of pain mediators, increased the innervation of sensory nerve fibers in the synovium, and worsened peripheral hyperalgesia. It also showed increased TRPV1 positive sensory innervation in KOA was mediated by NGF/TrKA signaling and exacerbated peripheral hyperalgesia.

## 1. Introduction

Knee osteoarthritis (KOA) is a common degenerative disease, and the inflammation of synovial tissue triggers nerve changes causing peripheral hyperalgesia in KOA which has received increasing attention. Chronic pain, as the main symptom of KOA, significantly affects the quality of life of patients worldwide. To date, there are no disease-modifying drugs or treatments that can stop or reverse the KOA process. Anatomically, KOA pain is caused by nociceptors, which are actually sensory nerve fiber endings that can detect stimuli to produce and transmit pain signals through dorsal root ganglia (DRG) to the posterior horn of the spinal cord, causing a cortical reflex to produce pain response. Nevertheless, the complex pathologic mechanisms of sensory innervation involved in KOA pain need further elucidation.

**Figure 1:**
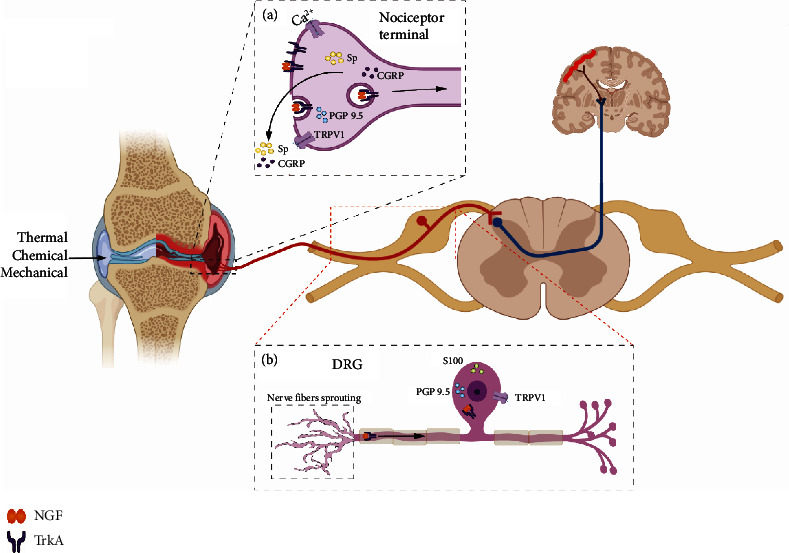
Relationship between NGF/TrKA signaling pathway and KOA peripheral hyperalgesia. *Notes*. (a) The activation of NGF/TrkA promotes the release of pain-related neuropeptides such as calcitonin gene-related peptide (CGRP) and substance P (SP) and activates TRPV1 on nociceptor terminal; NGF/TrKA complex is transported to DRG via cytokinesis. (b) The NGF/TrKA complex promotes DRG sensitivity and causes increased expression of TRPV1, PGP9.5, and S100, and it may also cause peripheral sensory nerve fiber sprouting.

Nerve growth factor (NGF) is considered closely related to inflammatory pain and neuropathic pain. As a potential target for pain treatments [1], it regulates neuronal growth and development and is important for sensory nerve innervation [2, 3]. NGF initiates signal transduction through its primary functional receptor tropomyosin receptor kinase A (TrKA) [4, 5]. The activation of NGF/TrkA recruits many signaling molecules that bind to the intracellular-phosphorylated tyrosine residues within TrkA by means of Src homology domains. At least two proteins have been identified that were recruited: phospholipase C gamma-1 (PLC*γ*1) and phosphatidylinositol-3-kinase (PI3K) [6–9]. These pathways eventually sensitize transient receptor potential vanilloid 1 (TRPV1) and cause changes in the fluidity of intracellular and extracellular Ca^2+^, resulting in increased sensitivity of tissues and organs [10]. The activation of NGF/TrkA also promotes the release of inflammatory mediators such as interleukin-1*β* (IL-1*β*) and pain-related neuropeptides and activates nociceptors to aggravate KOA pain [11] (Figure 1(a)). In a word, the NGF/TrKA signaling pathway can directly or indirectly regulate TRPV1 [12], which is considered closely related to inflammatory pain and neuropathic pain [13–16], and might regulate TRPV1 highly expressed in knee synovium, increasing nociceptor sensitivity and aggravating neuropathic pain [17].

Frontier researchers are attempting to find the link between NGF, sensory innervation, and KOA pain. They found the increased innervation in the symptomatic KOA synovial tissue, while the innervation and pain were decreased after using the NGF monoclonal antibody [18–20]. NGF monoclonal antibodies have demonstrated impressive analgesic effects; however, clinical studies have found it may cause unacceptable adverse effects such as rapidly progressive KOA, and therefore, intervention with TrKA, the main functional receptor for NGF, may have better prospects for application.

Does nerve fiber sprouting change sensory innervation in the KOA synovium and aggravate KOA pain (Figure 1(b))? Based on the above background, the main purpose of this study is to observe whether synovial nerve fiber sprouting aggravates KOA pain and how is it regulated by the NGF/TrKA signaling pathway.

## 2. Materials and Methods

### 2.1. Reagents

Lipopolysaccharide (LPS) and type I collagenase were purchased from Sigma-Aldrich Co. Ltd. (Sigma, MO, USA). Trizol, Dulbecco's modified Eagle culture medium (DMEM), penicillin-streptomycin mixture, and fetal bovine serum (FBS) were purchased from Gibco (Rockville, USA). The primary antibodies of *β*-actin, NGF, TrKA, TRPV1, IL-1*β*, and PGP 9.5 were purchased from Bioss (Beijing China). The Sirius red staining kit and goat anti-rabbit IgG H&L (HRP) kit were also purchased from Abcam (Cambridge, UK). 5 × HiScript II qRT SuperMix and 2 × Chamq SYBR qPCR MasterMix were purchased from Vazyme (Nanjing, China). Primers were supplied by Generay Biotechnology (Shanghai, China). ELISA kits for NGF and SP were purchased from JinyiBai Company (Nanjing, China).

### 2.2. In Vivo Animal Experimental Design and Ethics Statement

Animal experiments were conducted in accordance with the Chinese Guidelines for Animal Care and Welfare, and this study protocol was approved by the Animal Care and Use Committee of Nanjing University of Chinese Medicine (No. A210101).

Forty 8-week-old SD male rats, weighing 180–220 g, were obtained from Vital River Animal Technology (Beijing, China). The rats were raised in an SPF environment under a temperature of 18–22°C, a humidity of 28%, and light for 12 h/day. The rats were randomly divided into 4 groups at day 0 by using a randomized digital table: normal (*n* = 10), KOA (*n* = 10), KOA + NGF (*n* = 10), and KOA + siRNA TrKA (*n* = 10) after one-week adaptive feeding. The sample size was calculated by ANOVA assuming that *α* = 0.05 and power = 0.80. The experimental groups were established by the KOA model for the ACLT method [21], 3% pentobarbital sodium (100 mg/kg) was injected intraperitoneally for anesthesia, and 1 cm-long incision was made to expose and cut the anterior cruciate ligament and repeated at the other knee. Antibiotics (80,000 unit penicillin) were intramuscularly injected to prevent infection once a day, 3 days in total. On day 14, the KOA + NGF group was intra-articularly injected with recombinant human NGF-*β*, 2 *μ*g/joint once every two days, 14 days in total (7 injections); the KOA + siRNA TrKA group was intra-articularly injected with siRNA TrKA, 100 pmol/joint every two days, 14 days in total (7 injections); the normal group and the KOA group were injected with the same volume of 0.9% saline as control.

**Figure 2:**
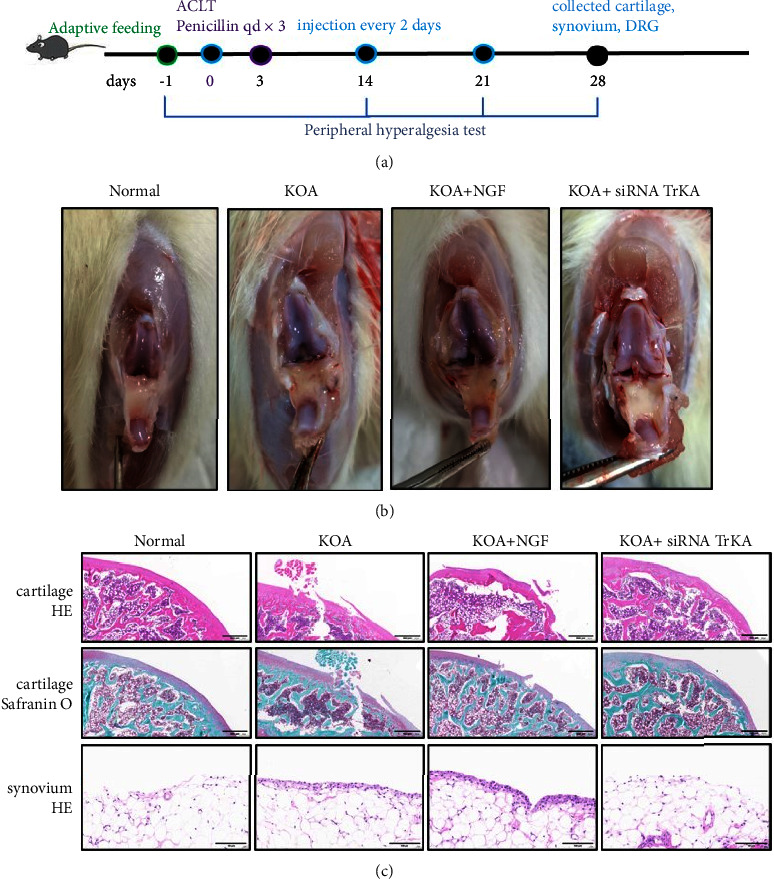
The protocol of animal experiment and the results of ACLT modeling. *Notes*. (a) Animal experimental protocol. (b) The anatomical changes in the knee. (c) HE (20x, scale bar = 500 *μ*m) and Safranin O‐Fast green staining (20x, scale bar = 500 *μ*m) of the cartilage and the HE staining of synovial tissues (40x, scale bar = 50 *μ*m).

All rats were sacrificed by intraperitoneal injection of 3% sodium pentobarbital (100 mg/kg), and the synovial tissue, cartilage, DRG, and serum were harvested on day 28 (Figure 2(a)).

### 2.3. Preparation and Stimulation of Primary Rat Fibroblast-Like Synoviocytes (FLSs) In Vitro

Synovial tissue was extracted from 6 to 8 weeks old SD male rats, washed twice with PBS, cut into 2-3 mm^2^ size, homogenized, digested in 0.2% type I collagenase, and incubated at 37°C for 4 h, filtered, centrifuged, and resuspended. 10 mL of 10% FBS and 1% penicillin-streptomycin solution were added to the Petri dish. The medium was changed every two or three days. The 3rd to 5th generations of FLS were used for experiments. FLS was randomly divided into normal, LPS, LPS + NGF, and LPS + siRNA TrKA groups. The LPS group was intervened with normal medium containing 5 *μ*g/mL LPS [22] for 24 h and then changed to a normal medium for 24 h. The LPS + NGF group was first treated with a normal medium containing 5 *μ*g/mL LPS for 24 h and then changed to a normal medium containing 20 *μ*M NGF-*β* for another 24 h [23]. The LPS + siRNA TrKA group was first treated with a normal medium containing 5 *μ*g/mL LPS for 24 h, then transfected with siRNA for 6 h, and changed to a normal medium for 18 h. Then, the FLS and supernatant were collected separately.

### 2.4. The Preparation and Stimulation of DRG In Vitro

DRG was extracted from the same SD rats, washed twice with PBS, put into mixed digestive enzymes, digested at 37°C for 1 h, filtered, centrifuged, and resuspended. Inoculation was performed in 35 mm Petri dishes, and 2 mL of the medium was added at 37°C. DRG neurons were divided into 4 groups corresponding to FLS and were intervened with the corresponding supernatant of the FLS culture medium for 24 h.

### 2.5. Pain Threshold Test

MWT was measured by the von Frey test (Institute of Biomedical Engineering, Chinese Academy of Medical Sciences, BME-404), referring to the method of Chaplan et al. [24], and the results were expressed in grams (g).

CWT was measured by using a temperature-adjustable metal plate system (35150-001, Ugo Basil SLR, Italy). Nociceptive responses to cold (4 ± 0.5°C) stimuli in the hind paws and the results were expressed in seconds(s).

### 2.6. Western Blot

All of the synovial tissue and L3-5 DRG tissues were harvested and lysed in RIPA, and the protein concentration was measured by the BCA protein assay kit. Protein samples were separated on 12% SDS-PAGE gels and then transferred to PVDF. PVDF was blocked with milk 2 h at room temperature, the primary antibody was added overnight at 4°C, and the secondary antibody was added for 2 h. The images of protein bands were collected and analyzed by using the ImageQuant LAS4000mini imaging system.

### 2.7. Quantitative Real-Time Polymerase Chain Reaction (qRT-PCR)

RNA was isolated from tissues and cells with Trizol, respectively. Reverse transcription was performed by using 5 × HiScript II qRT SuperMix. qPCR was performed using SYBR qPCR MasterMix on ABI PRISM 7300. The primer sequences are shown in Table 1.

### 2.8. Histology, Immunofluorescence Labeling, and Histological Analysis

The harvested cartilages and synovial tissues were fixed in 4% paraformaldehyde for 24 h, then decalcified in 10% EDTA, embedded in paraffin, sectioned, and stained with HE and Safranin O-Fast green. L3–5 DRG tissues were fixed in 4% paraformaldehyde for 6 h, dehydrated, and sectioned. The primary antibody was added and blocked, and then, the sections were incubated in the immunofluorescence antibody dilution buffer for 12 h at 4°C and incubated with the secondary antibody for 2 h at room temperature. Nuclear staining was performed with DAPI and then observed with a fluorescence microscope. Three sections were taken from each group to observe the changes in synovial tissues and DRG tissues. Image analysis was performed with ImageJ software.

### 2.9. Statistical Analysis

Data are presented as mean ± standard deviation. Statistical analysis was performed with Graphpad Prism 8.0 software (San Diego, CA, USA) via ANOVA and two-way ANOVA, and *P* < 0.05 (two tailed) was considered as statistically significant.

## 3. Results

### 3.1. The Results of ACLT Modeling

First of all, we examined the modeling results. The anatomical changes showed the cartilage in the KOA group was uneven, thin, and worn, these changes were even more in the KOA + NGF group, and these changes were improved in the KOA + siRNA TrKA group (Figure 2(b)). HE and Safranin O-Fast green staining showed the cartilage in the KOA group and KOA + NGF group was worn and ruptured, the trabecular bone was disordered compared with that in the normal group, and the cartilage changes in the KOA + siRNA TrKA group were less. The synovial HE staining in the KOA group showed synovial cells proliferated, were arranged disorderly, and were infiltrated with more inflammatory cells compared with those in the normal group; these changes were improved in the KOA + siRNA TrKA group (Figure 2(c)).

### 3.2. NGF/TrKA Modulates Peripheral Hyperalgesia and the Expression of Neural Markers in Synovial Tissues

**Figure 3:**
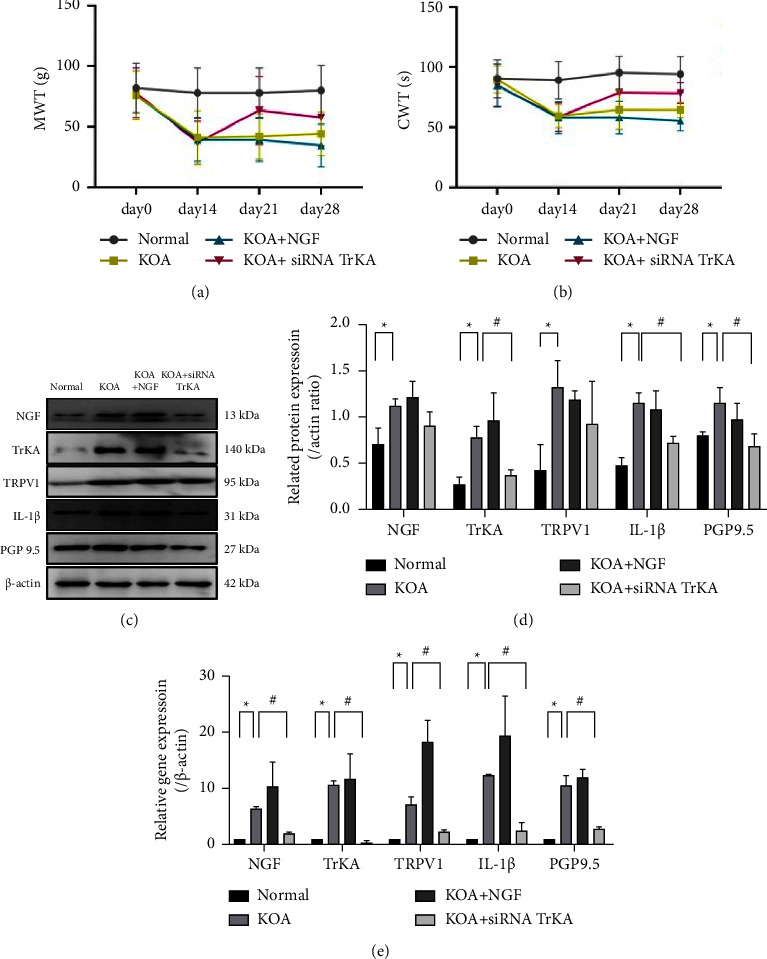
NGF-induced peripheral hyperalgesia and related protein and gene expressions in synovial tissues. *Notes*. (a, b) The peripheral hyperalgesia thresholds. (c, d) The protein expressions in synovial tissues. (e) The gene expressions in synovial tissues. ^*∗*^*P* < 0.05 between the normal group and the KOA group. ^#^*P* < 0.05 between the KOA group and the KOA + siRNA TrKA group.

We measured the MWT and CWT of all rats on day 0, day 14, day 21, and day 28. The MWT and CWT in the KOA group decreased compared with those in the normal group on day 14 (*P* < 0.05), and they continued decreasing after the injection of NGF; meanwhile, the MWT and CWT increased after the injection of siRNA TrKA compared with the KOA group (Figures 3(a) and 3(b)).

The western blot results showed there was a significant increase in the protein expressions of NGF, TRPV1, IL-1*β*, and PGP9.5 in the KOA group compared with the normal group (^*∗*^*P* < 0.05); these protein expression trends were similar in the KOA + NGF group; the protein expressions decreased in different degrees in the KOA + siRNA TrKA group (^#^*P* < 0.05) (Figures 3(c) and 3(d)).

The qRT-PCR results showed there was a significant increase in the gene expressions of NGF, TrKA, TRPV1, IL-1*β*, and PGP9.5 in the KOA group (^*∗*^*P* < 0.05), and these gene expressions were even higher in KOA + NGF group; these gene expressions were significantly decreased in the KOA + siRNA TrKA group compared with those in the KOA group (^#^*P* < 0.05) (Figure 3(e)).

### 3.3. NGF/TrKA Increased TRPV1-Labeled Sensory Nerve Fibers in Synovial Tissues

**Figure 4:**
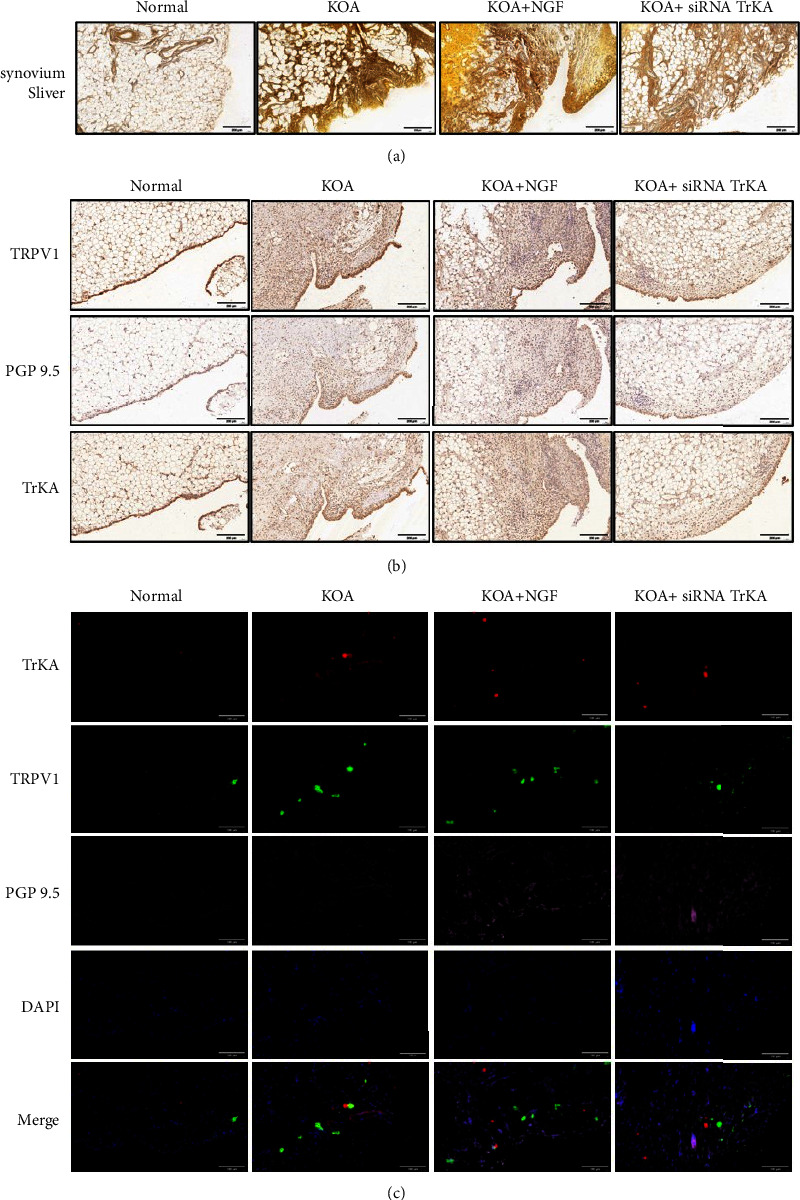
NGF-induced TRPV1-labeled nerve fiber sprouting. *Notes*. (a) The silver staining images of synovial tissues (20x, scale bar = 200 *μ*m). (b) The immunohistochemical staining images of synovial tissues, in which the target protein turns to brown particles after staining (20x, scale bar = 200 *μ*m). (c) The immunofluorescence staining images of TrKA, TRPV1, and PGP9.5 in the synovial tissues (40x, scale bar = 100 *μ*m).

The color of silver staining in synovial tissues was deeper, and the positive area of nerve fibers increased in the KOA group than that in the normal group; the color was lighter, and the positive area was decreased in the KOA + siRNA TrKA group (Figure 4(a)).

The immunohistochemical staining images of synovial tissues showed the positive expressions of PGP9.5, TrKA, and TRPV1 in the KOA group were higher than those in the normal group. This trend was more obvious in the KOA + NGF group, and these positive expressions decreased in the KOA + siRNA TrKA group (Figure 4(b)).

To observe the causes of NGF-induced peripheral hyperalgesia and whether they were regulated by the NGF/TrKA signaling pathway, we performed immunofluorescence staining on synovial tissues. We observed the positional relationship among TrKA, TRPV1, and PGP 9.5 by immunofluorescence in the synovial tissues, the results showed that TrKA, TRPV1, and PGP9.5 were colocated in each group (Figure 4(c)). We also observed the positional relationship among NGF, TRPV1, and PGP 9.5, and the results showed NGF, TRPV1, and PGP9.5 were two-by-two colocalized in the synovial tissues in each group.

### 3.4. NGF/TrKA Induced the Upregulation of TRPV1 and Neural Markers in DRG

**Figure 5:**
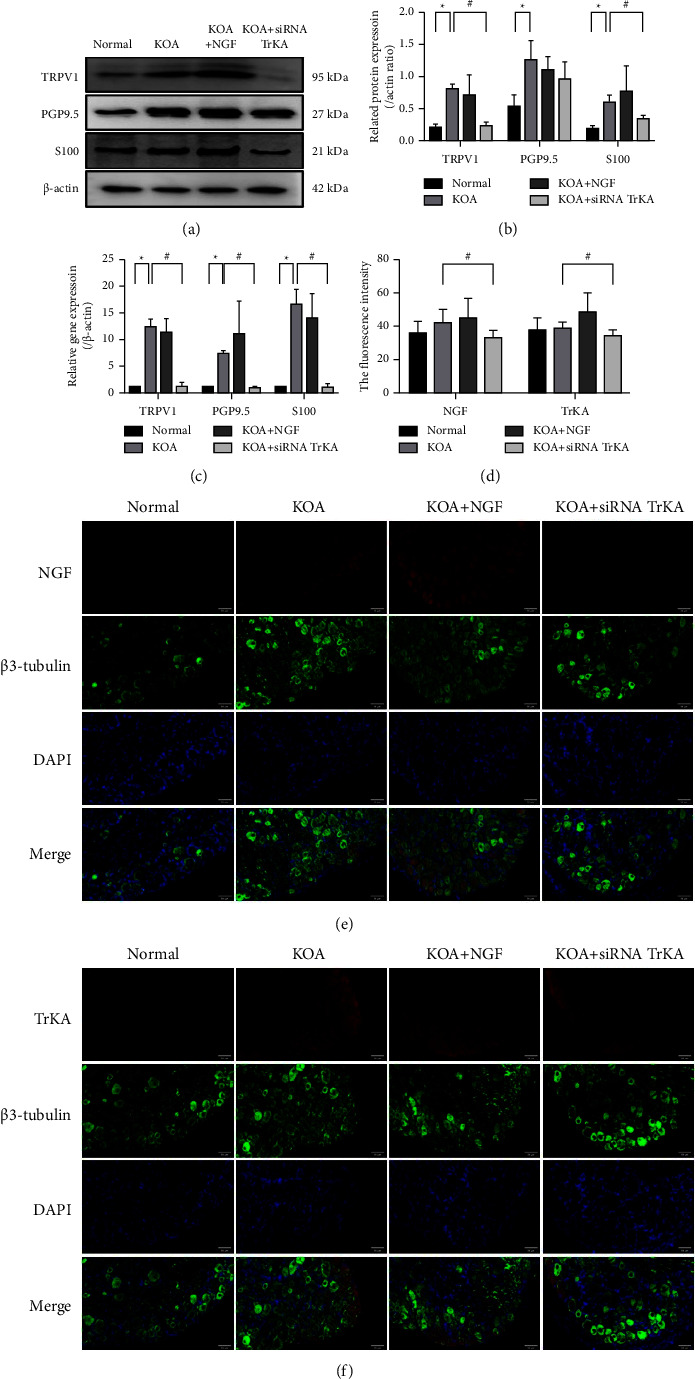
The related protein and gene expressions in DRG. *Notes*. (a, b) The protein expressions in DRG tissues. (c) The gene expressions in DRG tissues. (d) The fluorescence intensity of NGF and TrKA in DRG. (e, f) NGF and TrKA were colocalized to *β*3-tubulin, respectively (40x, scale bar = 50 *μ*m). ^∗^*P* < 0.05 between normal and the KOA group. ^#^*P* < 0.05 between the KOA and the KOA + siRNA TrKA group.

We detected the protein and gene expressions of pain-related TRPV1 and pan-neural markers PGP 9.5 and S100 in DRG tissues. There was a significant increase in the protein expressions of TRPV1, PGP9.5, and S100 in the KOA group and KOA + NGF group compared with those in the normal group (^*∗*^*P* < 0.05), while the protein expressions of TRPV1 and S100 were significantly decreased in the KOA + siRNA TrKA group compared with those in the KOA group, and yet, there was no significant difference in the protein expression of PGP9.5 (^#^*P* < 0.05) (Figures 5(a) and 5(b)). There was a significant increase in the gene expressions of TRPV1, PGP 9.5, and S100 in the KOA group and KOA + NGF group compared with those in the normal group (^*∗*^*P* < 0.05), while these gene expressions significantly decreased in the KOA + siRNA TrKA group (^#^*P* < 0.05) (Figure 5(c)).

The immunofluorescence of NGF and TrKA in DRG tissues was also observed. The fluorescence intensity of NGF and TrKA was increased in the KOA and KOA + NGF group compared with that in the normal group, and the fluorescence intensity decreased in the KOA + siRNA TrKA group (^#^*P* < 0.05) (Figure 5(d)). NGF and TrKA were colocalized to *β*3-tubulin, which is also a neural marker (Figures 5(e) and 5(f)).

### 3.5. NGF/TrKA Involved in LPS-Induced TRPV1-Related Changes in FLS

**Figure 6:**
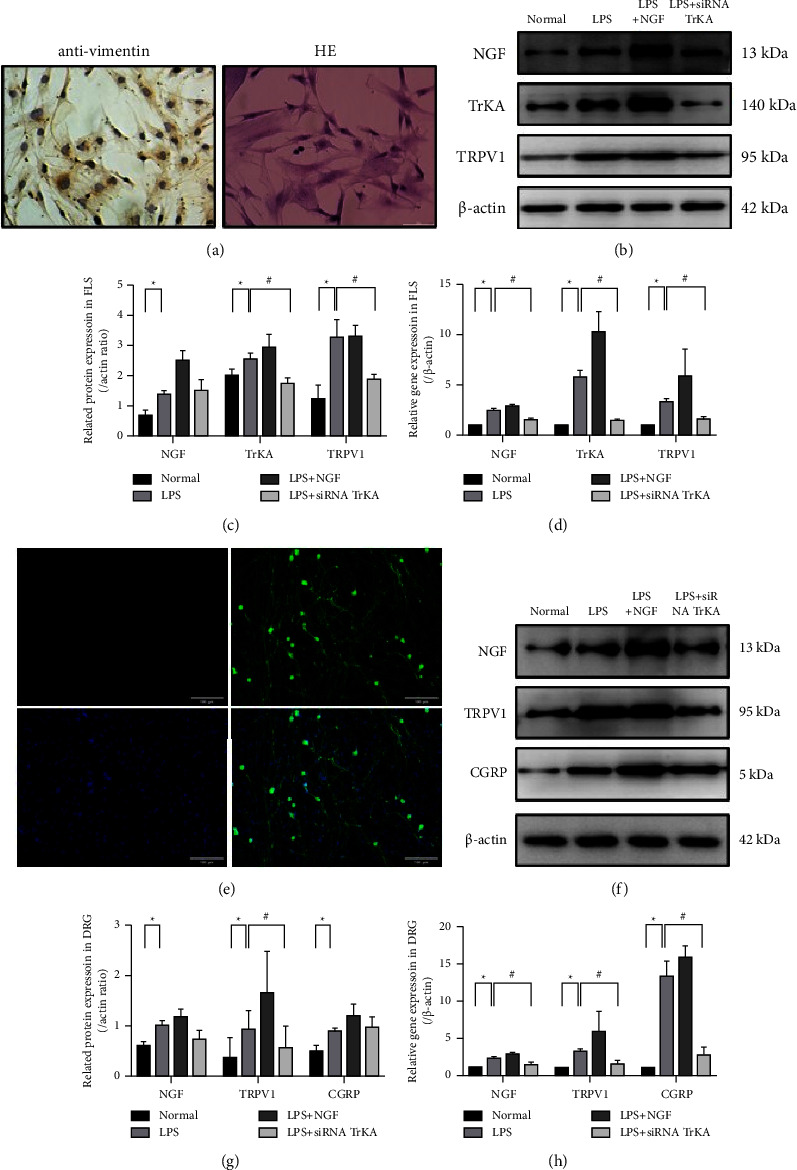
The protein and gene expressions in FLS and DRG neurons. *Notes*. (a) FLS was identified by antivimentin immunostaining and HE staining (40x, scale bar = 50 *μ*m). (b, c) The protein expressions in FLS. (d) The gene expressions in FLS. (e) DRG neurons were identified by GFAP (red) and *β*3-tubulin (green) immunostaining (40x, scale bar = 100 *μ*m). (f, g) The protein expressions in DRG neurons. (h) The gene expressions in DRG neurons. ^*∗*^*P* < 0.05 between the normal group and the LPS group, ^#^*P* < 0.05 between the LPS group and the LPS + siRNA TrKA group.

First, FLS was identified by antivimentin immunostaining and HE staining before the experiment (Figure 6(a)). Next, we verified the above results with FLS. The results showed there was a significant increase in the protein expressions of NGF, TrKA, and TRPV1 in the LPS and LPS + NGF group (^*∗*^*P* < 0.05), and these protein expressions decreased at different degrees in the LPS + siRNA TrKA group (^#^*P* < 0.05) (Figures 6(b) and 6(c)). There was also a significant increase in the gene expressions of NGF, TrKA, and TRPV1 in the LPS and LPS + NGF group (^*∗*^*P* < 0.05), and these gene expressions significantly decreased in the LPS + siRNA TrKA group (^#^*P* < 0.05) (Figure 6(d)).

### 3.6. NGF/TrKA Affects the Expressions of TRPV1 and CGRP in DRG Neurons

Similarly, DRG neurons were identified by GFAP and *β*3-tubulin immunostaining before the experiment (Figure 6(e)). The results showed there was a significant increase in the protein expressions of NGF, TRPV1, and CGRP in the LPS and LPS + NGF group compared with those in the normal group; there was also a significant decrease of TRPV1 in the LPS + siRNA TrKA group compared with the LPS group (^*∗*^*P* < 0.05); yet, there was no significant difference of NGF and CGRP between the LPS + siRNA TrKA group and the LPS group (Figures 6(f) and 6(g)). There was a significant increase in gene expressions of NGF, TRPV1, and CGRP in the LPS and LPS + NGF group compared with those in the normal group (^*∗*^*P* < 0.05), and these gene expressions also significantly decreased in the LPS + siRNA TrKA group (^#^*P* < 0.05) (Figure 6(h)).

## 4. Discussion

Chronic pain has been an important burden for KOA patients, especially in advanced KOA patients, and KOA patients typically live with this chronic pain for about 30 years after diagnosis [25]. There is still a lack of understanding of synovial sensory nerve sprouting, which may play an important mediating role in peripheral hyperalgesia. Therefore, further research is needed, and it is also useful for clinical decision. The purpose of this study is to observe that the mechanism of the NGF/TrKA signaling pathway regulates peripheral hyperalgesia and whether peripheral hyperalgesia could be improved by inhibiting TrKA. There are three main methods widely used [26]: monoiodoacetate (MIA), destabilization of medial meniscus (DMM), and ACLT. Zhang et al. [21] found that pain mediators increased and peripheral hyperalgesia thresholds decreased in all rats by these three modeling methods. They also found nerve fiber sprouting in KOA synovium and verified that the ACLT method can better simulate the pathological changes in human KOA.

In this study, we found that the peripheral hyperalgesia thresholds decreased in the KOA and the KOA + NGF group, while these thresholds were improved after intra-articular injection of siRNA TrKA to inhibit the NGF/TrKA signaling pathway. The protein and gene expressions of inflammatory factors (e.g., IL-1*β*), pain mediators (e.g., NGF and TRPV1), and neural markers (e.g., PGP9.5) were increased in KOA synovial tissues, and these protein and gene expression changes increased even higher in the KOA + NGF group; however, the expressions of these factors decreased in the KOA + siRNA TrKA group. Then, we found that the protein and gene expressions trends of TRPV1, PGP9.5, and S100 in DRG tissues were similar to those in synovial tissues.

Next, we verified the synovial innervation of sensory nerve fibers by silver staining and immunohistochemistry staining. Silver staining is mainly for nerve fiber density. PGP9.5 is a pan-neural marker that is present in all afferent and efferent nerve fibers, is specific to nerve fibers, and is often used as an axonal marker. The positive area of silver staining and PGP9.5 of immunohistochemistry staining were increased in the KOA and the KOA + NGF group compared with those in the normal group; it indicated nerve fiber sprouting. The positive area of silver staining and PGP9.5 immunohistochemistry were decreased in the KOA + siRNA TrKA group as expected. In further research, the NGF and TrKA immunofluorescence in synovial tissues colocalized with TRPV1 and PGP9.5. Meanwhile, the fluorescence intensity of TRPV1 and PGP9.5 in synovial tissues decreased in the KOA + siRNA TrKA group compared with that in the KOA group. It indicated that some of the newly sprouting nerve fibers were TRPV1-labeled, and this pathological process is regulated by TrKA.

The fluorescence of NGF and TrKA was co-localized with *β*3-tubulin, respectively, in DRG tissues and increased in the KOA and the KOA + NGF group and decreased in the KOA + siRNA TrKA group. It indicated that NGF might intervene DRG by forming the NGF/TrKA complex and then transporting to DRG via cytokinesis, and this process was regulated by TrKA.

FLS and DRG neurons were divided into four groups correspondingly as in vivo. After induction of the FLS inflammation model with LPS, the protein and gene expressions of NGF, TrKA, and TRPV1 in FLS were significantly increased in the LPS group, this increased expressive trend was further in the LPS + NGF group, and this increased expressive trend was partly reversed after intervened with siRNA TrKA. Next, we intervened the DRG neurons with the FLS supernatant to simulate the coculture with FLS. The protein and gene expressions of NGF, TRPV1, and CGRP were significantly increased in the LPS group and LPS + NGF group compared with those in the normal group, and there were also significantly decreased protein and gene expressions of NGF, TRPV1, and CGRP in the LPS + siRNA TrKA group compared with those in the LPS group. This is similar to the results in vivo, demonstrating that inflammation can activate the NGF/TrKA signaling pathway and promote the release of pain mediators, and this progress may be regulated by TrKA.

Kc et al. [27] found the degree of cartilage damage was significantly improved, but peripheral hyperalgesia was increased in the PKC-*δ* knockout KOA rats, and attributed this contradiction to the increased expressions of NGF, TrKA, and PGP9.5 in the synovium. They pointed out that the inhibition of PKC-*δ* perhaps activated the NGF/TrKA signaling pathway, which may further promote sensory nerve fiber sprouting, increasing peripheral hyperalgesia. This result is similar to our study, the nerve fiber innervation in KOA synovium was significantly increased, and it might be regulated by the NGF/TrKA signaling pathway. Peripheral hyperalgesia can be regulated by the NGF/TrKA signaling pathway. On the one hand, the activation of the NGF/TrKA signaling pathway increased the expression of inflammation and pain mediators, and on the other hand, the NGF/TrKA signaling pathway promoted the innervation in the synovium.

Zhang et al. [21] found the density of sensory nerves increased in all rats modeled by MIA, DMM, and ACLT, and the expressions of NGF were upregulated. They also suggested sensory nerve sprouting in the KOA synovium was strongly correlated with KOA itself rather than the causes of KOA. Neuropathic pain was also related to TrKA and TRPV1 [28]; using the TrKA antibody can reduce the innervation of nerve fibers [18]. All of these findings indicate nerve fiber sprouting was correlated with the NGF/TrKA signaling pathway and perhaps not be limited to the synovial tissue but also spread to the subchondral bone.

Which kind of nerve fibers will deteriorate the peripheral hyperalgesia in KOA? The results of this study showed that the fluorescence of TRPV1 was increased in the KOA synovium and the binding of NGF and TrKA upregulated TRPV1 positive sensory nerve fiber sprouting. This sprouting possibly increased the sensing area of stimuli in the synovium and deteriorated the local pain. The NGF/TrKA complex enters DRG and activates TRPV1 there and increases the excitability of DRG so that it is easily activated by noninjurious signals and forms peripheral hyperalgesia. In short, the high level of NGF increased the nerve fiber innervation of synovium, especially TRPV1-labeled sensory nerve fibers, and deteriorated peripheral hyperalgesia. The above pathological changes were regulated by TrKA so that they were partially reversed and peripheral hyperalgesia improved after inhibiting TrKA.

Clinical trials with NGF monoclonal antibodies, which broadly inhibit the function of NGF and were found to cause rapidly progressive KOA, were once called off. Research on its functional receptor TrKA may facilitate further development of the potential to inhibit NGF for the treatment of chronic pain and benefit KOA patients.

## 5. Conclusion

In conclusion, this study showed that the activation of the NGF/TrKA signaling pathway in KOA promoted the release of pain mediators and inflammatory factors, increased the innervation of sensory nerve fibers in the synovium, and worsened peripheral hyperalgesia, and the above pathological changes could be partially reversed by inhibiting TrKA. There are [29] still some limitations in this study due to the research conditions, such as not being able to set TrKA knockout or overexpression group, and not being able to quantify sensory nerve sprouting, which will be further improved in future studies.

## Figures and Tables

**Table 1:** Primer sequences in qRT-PCR.

Genes	Forward primer (5′-3′)	Reverse primer (5′-3′)
NGF	CCTCTTCGGACACTCTGG	CTCCAACCCACACACTGA
TrKA	TGGGAGCAGGAGGATTTG	TGGGCATCTGGATCTTCAC
TRPV1	CCTTCTGCTCAACATGCTC	GCCTTCCTCATGCACTTC
IL-1*β*	ATGGTCGGGACATAGTTGA	CTTGGCAGAGGACAAAGG
CGRP	TCCTGGTTGTCAGCATCTT	AGGCGAGCTTCTTCTTCAC
PGP 9.5	AGCTGGAATTTGAGGATGGA	ACTTGGCTCTGTCTTCAGG
S100	GGGACAAATATAAGCTGAGCA	ATCCTTCTGGACATCCAGG
*β*-Actin	GAGAGGGAAATCGTGCGT	GGAGGAAGAGGATGCGG

## Data Availability

The data used to support the findings of this study are available from the corresponding author upon request (supplementary files S1, S2, and S3).

## Abbreviations

KOA: Knee osteoarthritis
SPF: Specific pathogen free
SD: Sprague-Dawley
LPS: Lipopolysaccharide
NGF: Nerve growth factor
PLC*γ*1: Phospholipase C gamma-1
DAG: 1,2-diacylglycerol
IL-1*β*: Interleukin-1*β*
TNF-*α*: Tumor necrosis factor-*α*
TrKA: Tropomyosin receptor kinase A
TRPV1: Transient receptor potential vanilloid 1
FLS: Fibroblast-like synoviocytes
NSAIDs: Nonsteroidal anti-inflammatory drugs
DRG: Dorsal root ganglia
CGRP: Calcitonin gene-related peptide
SP: Substance P
PGP 9.5: Protein gene product 9.5
ACLT: Anterior cruciate ligament transection
PCR: Polymerase chain reaction
MWT: Mechanical withdrawal threshold
CWT: Cold withdrawal threshold.

## Additional Points

*Perspective*. High levels of NGF led to peripheral hyperalgesia in KOA, but whether it was associated with increased sensory innervation remains unclear. NGF monoclonal antibodies have been extensively studied and have demonstrated impressive analgesic effects, but clinical studies have found it may cause adverse effects such as rapidly progressive KOA, and therefore, intervention with TrKA, a functional receptor for NGF, may have better prospects for application. This study provides evidence for the potential mechanism of KOA sensory innervation involved in peripheral hyperalgesia, and it is modulated by TrKA and provides a potential target for clinical treatments of KOA pain.

## Supplementary Materials

Supplementary Materials S1: author checklist: obtain the 2-page ARRIVE full checklist. S2: CONSORT 2010 Checklist. S3: statistical analysis: *P* value, *P* means, and SDs, etc.
