# Supplementary material for: NGF Signaling Exacerbates KOA Peripheral Hyperalgesia via the Increased TRPV1-Labeled Synovial Sensory Innervation in KOA Rats
Source: Pain Res Manag. 2024 Feb 19;2024:1552594. doi: 10.1155/2024/1552594 (PMC10896652; doi:10.1155/2024/1552594)
Supplement: Supplementary Materials — S1: author checklist: obtain the 2-page ARRIVE full checklist. S2: CONSORT 2010 Checklist. S3: statistical analysis: P value, P means, and SDs, etc. [file 1552594.f1.zip › S3 Statistical Analysis P value (1).docx]

**synovial tissue WB**

**synovial tissue PCR**

**DRG tissue WB**

**DRG tissue PCR**

**FLS WB**

**FLS PCR**

**DRG cell WB**

**DRG cell PCR**

**fluorescence intensity**
